# Gender Differences in Psychosocial Outcomes of Hair Loss Resulting from Childhood Irradiation for Tinea Capitis

**DOI:** 10.3390/ijerph18157825

**Published:** 2021-07-23

**Authors:** Liat Hoffer, Netta Achdut, Shifra Shvarts, Dorit Segal-Engelchin

**Affiliations:** 1 Faculty of Health Sciences, Ben-Gurion University of the Negev, POB 653, Beer-Sheva 84105, Israel; Liathof@post.bgu.ac.il (L.H.); shvarts@bgu.ac.il (S.S.); 2 The Spitzer Department of Social Work, Ben-Gurion University of the Negev, POB 653, Beer-Sheva 84105, Israel; nettaach@bgu.ac.il

**Keywords:** tinea capitis, childhood irradiation, hair loss, psychosocial symptoms, depression, gender differences

## Abstract

Recent studies have linked hair loss due to childhood irradiation for tinea capitis, a fungal infection of the scalp, to adverse psychosocial and health outcomes in women. However, no study to date has examined gender differences in the outcomes of this type of hair loss. The current study aimed to investigate gender differences in health and psychosocial outcomes of hair loss resulting from childhood irradiation for tinea capitis, and to identify the risk factors associated with depression in both men and women. Medical records held at the archives of the Israel National Center for Compensation of Scalp Ringworm Victims were retrospectively reviewed for 217 women and 105 men who received maximum disability compensation due to severe hair loss resulting from irradiation for tinea capitis. We found that women were at increased risk of developing psychosocial symptoms, including depression. Gender emerged as a significant predictor of depression, distinct from other predictors, such as marital status, age at radiation, exposure to verbal and physical bullying, low self-esteem, social anxiety, and physical health problems. Thus, the psychosocial needs of patients, particularly female patients, who were irradiated for tinea capitis during childhood need to be taken into account by the healthcare professionals treating them.

## 1. Introduction

Although both men and women cope with hair loss caused by diseases and medical treatments, relatively little scholarly attention has been given to men’s experiences of hair loss [1,2,3,4]. This may be due to the assumption that hair loss is more of a problem for women [2,5], who place more importance on their appearance than men [5,6] and for whom hair represents a significant aspect of their identity [7,8] and is perceived as a major indicator of reproductive potential, femininity, sexuality, and attractiveness in society [7,9,10]. However, studies focusing on men with androgenetic alopecia, also known as male pattern hair loss or common baldness in men [11], affecting up to 80% of men by old age [12], indicate that hair loss can also be a significant problem for men [5]. Despite being perceived by clinicians as a mild dermatological condition [13], men with androgenetic alopecia report a high degree of concern about their hair loss [14,15], including reduced self-confidence in personal attractiveness, negative impacts on social life [16], depression [16,17], decreased quality of life (QoL) [5,13], low self-esteem, and high anxiety levels compared to their counterparts [17,18]. The negative impact of hair loss on men was also documented in a recent study of male breast cancer patients. This study showed that men experience the loss of their hair, particularly the loss of their beard, which is a marker of masculinity, as interfering with their social interactions because it identifies them as being sick [2].

Research on gender differences in the psychosocial effects of hair loss has also been limited, yielding mixed findings [19]. Some studies have indicated that men and women are similarly affected by hair loss. For example, a recent qualitative study investigating experiences of chemotherapy-induced hair loss among men and women with breast cancer revealed that hair loss is a distressing experience for both genders, affecting their identities and relationships [4]. Paterson et al.’s [3] systematic review of studies exploring the unmet supportive care needs of men and women with chemotherapy-induced hair loss showed that both men and women experience high levels of distress, altered body image, negative feelings toward hair loss, and limited social interactions due to their need to conceal hair loss. An earlier qualitative study of young men and women coping with chemotherapy-induced hair loss found that they both encounter difficulties adjusting to their altered image, and that they felt that the loss of hair exposed their illness [1]. Gender similarities in QoL have also been reported among Chinese patients with alopecia areata and androgenetic alopecia [20].

However, other studies have shown gender differences in the impact of hair loss. For example, Rosman [21] found that chemotherapy-induced alopecia is a more traumatic experience for women than for men; women regarded hair loss as a loss of vitality, strength, and health, whereas men viewed hair loss as a logical consequence of chemotherapy. Differences between men and women’s experiences of chemotherapy-induced alopecia were also reported by Can et al. [22], who found lower psychological well-being among women compared to men regardless of the severity of hair loss. Cartwright et al. [23], in a study of individuals with four types of alopecia (alopecia areata, alopecia universalis, alopecia totalis, and alopecia androgenetic), found lower QoL among women compared to men. Women were also more likely to report that the hair loss impacted their leisure activities and personal relationships, whereas men were more likely to report that hair loss affected their work. In a study focusing on individuals with androgenetic alopecia [24], depression was more common among women, whereas anxiety and aggressiveness were more common among men.

Individuals with a childhood history of irradiation for tinea capitis, which is a fungal infection of the scalp primarily affecting children [25,26], represent another population warranting investigation of the effects of hair loss among both men and women. Until the introduction of the antifungal griseofulvin in 1958, approximately 200,000 children worldwide [27], mainly in the United States, Europe, and the Middle East [28,29], were treated for tinea capitis by irradiation to induce epilation. Although hair loss is one of the commonly reported adverse effects of this treatment [27,30,31] and may also be an outcome of the type of tinea diagnosis (tinea favosa) [30], scientific research did not pay adequate attention to its psychosocial impacts. Two recent studies specifically investigated the health-related and psychosocial impacts of hair loss in women irradiated for tinea capitis during childhood. The first study [32] found that hair loss due to childhood irradiation for tinea capitis in women is linked to psychosocial impacts, including depression, anti-depressant and/or anti-anxiety drug use, psychiatric hospitalization, suicide attempts, social anxiety, and divorce, as well as health impacts, such as migraines, which have been found to be associated with childhood trauma and stressful life events in adulthood [33,34]. The second study [35] extended these findings, linking hair loss severity in women to an increased risk of mental health problems and showing an indirect effect of hair loss severity on mental health via an impact on women’s social life. To date, no study has investigated the effects of hair loss in men irradiated for tinea capitis during childhood or examined gender differences in the outcomes of hair loss resulting from childhood irradiation for tinea capitis.

The aims of the present study were to examine gender differences in health and psychosocial outcomes of hair loss due to childhood irradiation for tinea capitis, identify the risk factors associated with depression in both men and women irradiated for tinea capitis, and determine whether gender differences in depression risk are due to a higher prevalence of risk factors for depression among women or to the effect of gender per se.

## 2. Materials and Methods

### 2.1. Participants

Between 1995 and 2015, 4033 women and 4011 men received the maximum disability compensation (15–20% disability under the National Insurance criteria for alopecia) by the State of Israel’s medical committees due to significant hair loss resulting from childhood irradiation for tinea capitis [36]. The compensation was given to these individuals under Article 77 of Israel National Insurance Ordinances related to alopecia universalis [37]. Approximately 1164 of the women and 1160 of the men had medical records containing detailed information on their physical and mental health. This study is based on a sample of 322 medical records (*n* = 217 women, 67.4%; *n* = 105 men, 32.6%). The medical records were selected by a stratified sampling, with the strata determined by the year in which the compensation claims were filed (1995–2015). We randomly selected 16–17 detailed medical records from each of the twenty years studied here. Following Faul et al. [38], we conducted post-hoc power analyses using G* power 3 software to estimate the observed power based on the following parameters: α = 0.05, sample size = 322, multivariate logistic regression, odds ratio of 1.68, and R^2^ of 0.14 (14%) [39]. The analysis indicated a 92.44% chance of detecting gender differences in depression. Bujang et al. [40] propose sample size guidelines for multivariate regressions. They suggest a minimum sample size of 300 to generate a close approximation of estimates with the parameters in the population and contend that this is particularly necessary for non-experimental data, which is the type of data used in this study. In addition, the minimum required sample size was guided by the ratio between the number of independent variables and the number of cases. However, recommendations widely vary across studies that focus on the determination of this ratio, ranging from 10:1 to 30:1 in the context of multivariate regression [40]. The ratio in this study, based on our multivariate analysis, is considered to be good (1:23).

### 2.2. Measures

Data were retrieved from the medical records in accordance with ethical standards for such work as set forth in Article 20 (A) (7) of Israel’s 1996 Patient’s Rights Law [41]. The study was approved by the Human Subjects Research Committee of Ben-Gurion University of the Negev (BGU HSR 1843-1) and by the Helsinki Committee of Sheba Medical Center–Ramat Gan (SMC-2448-15—Helsinki Sheba).

Data retrieval focused on the following variables: demographic characteristics (i.e., age at filing the compensation claim, place of birth, marital status, education, employment), irradiation treatment (i.e., place of radiation, radiation age), self-reported exposure to physical and/or verbal bullying because of hair loss and period of exposure (childhood/adulthood/both periods), physician diagnosis of physical health conditions (e.g., migraines, hypertension, diabetes, cancer), and psychosocial conditions (e.g., self-reported low self-esteem; self- and professional-reported depression, social anxiety, and social avoidance). Professional reports of depression, social anxiety, and social avoidance were observed in approximately 80%, 48%, and 25% of the medical records in which these conditions were reported, respectively. Preliminary analysis showed no significant differences between participants with self-reported psychosocial problems (e.g., depression, social anxiety, and social avoidance) and those with professional reports of these psychosocial problems in regards to demographic characteristics and variables related to the irradiation treatment, including age at the time of irradiation. Therefore, we pooled self and professional reports for each of these three psychosocial conditions into one category and created a dummy variable for each of the three psychosocial conditions, whereby participants coping with these conditions received the value 1.

We conducted a Promax exploratory factor analysis (EFA) [42] for the socioeconomic measures of education and employment. This analysis yielded a factor variable (eigenvalues = 1.245) that contains both measures of employment status (loading = 0.789) and education level (loading = 0.789). The cumulative variance explained by this factor was 62.27%. Factor scores were calculated for each individual respondent using least squares regression, which has the highest validity of the factor scoring techniques [43]. Higher scores in this factor indicate higher socioeconomic status.

### 2.3. Data Analysis

First, we examined gender differences in the study variables. Differences in categorical variables were calculated using chi-squared tests for heterogeneity and trend. Differences between means were calculated using *t*-tests. Given the high prevalence of depression reported in the overall sample (56.2%) and the large gender difference that emerged in depression risk (see Table 1), we chose to focus on depression as the central indicator of the psychosocial consequences reported in the medical records.

Next, we conducted bivariate analyses to examine the associations of background characteristics, exposure to physical and verbal violence, psychosocial conditions, and physical health condition with depression. Differences in the probability of depression by categorical variables were calculated using chi-squared tests for heterogeneity and trend. Differences between means were calculated using *t*-tests.

We also employed five hierarchical logistic models. In the first step, we included the background characteristics detailed in Table 1 (model 1). In the second step, we added the variables related to exposure to verbal and physical bullying (model 2). However, due to small cell constraints, we omitted the period of exposure. In the third step, we added the psychosocial measures low self-esteem, social anxiety, and social avoidance (model 3). In the fourth step, the four physical health measures were added (model 4). In the fifth step, we incorporated the gender variable to examine its distinct contribution to depression beyond the contribution of background characteristics, exposure to bullying, psychosocial problems, and physical health problems (model 5). Lastly, we examined whether the role of bullying and psychosocial and health measures in the development of depression vary by gender (i.e., moderation effect). For this purpose, we computed two-way interaction terms between gender and each of the measures listed above. The dependent variable in all analyses was depression, divided into two categories: no depression (score = 0) and depression (score = 1). Data were analyzed using IBM SPSS Statistics 26 for Windows.

## 3. Results

### 3.1. Bivariate Analyses of the Study Variables

Table 1 presents the bivariate analyses of the study variables in the overall sample and by gender. The mean age of participants at the time of filing the compensation claim was 57.99 ± 7.02 years. Most participants were married (71.4%), engaged in paid work at the time of the claim (74.8%), and had low education levels (mean 7.95 ± 4.07 years of schooling). At the time of irradiation for tinea capitis, the mean age was 8.38 ± 3.39 years, with most participants (47.5%) being irradiated between 6 and 10 years of age.

Close to half of participants (45%) reported being exposed to verbal bullying because of hair loss during their lifetime. Of these, 60.7% reported having suffered from verbal bullying during childhood. Physical bullying due to hair loss was much less prevalent (7%). Therefore, we excluded the period of exposure to physical bullying from the analysis. Regarding psychosocial indicators related to hair loss, the percentage of participants who reported low self-esteem was extremely high (77.6%). Relatively high rates of self-reported and/or professional-reported social anxiety (29.5%) and social avoidance (40.7%) were observed. Depression was also noted in more than half of the medical records (56.2%). Lastly, physical health measures indicated an extremely high prevalence of migraines (71.7%) and tumors (40.4%).

Significant gender differences were found in several measures. Male participants had a higher level of education, and were more likely to be married, employed, and irradiated in Israel. Mean age at irradiation was higher for men than for women (9.16 ± 3.30 years and 8.03 ± 0.37 years, respectively). Low self-esteem was more prevalent among women compared to men (82.5% and 67.6%, respectively), similar to social anxiety (37.3% and 13.3%, respectively) and social avoidance (46.1% and 29.5%, respectively). Furthermore, women were at much higher risk of depression than men (66.8% compared to 34.3%, respectively). Among the physical health indicators, a significant difference was found only with respect to hypertension: 22.6% among women compared to 10.5% among men.

### 3.2. Bivariate Analyses of the Study Variables by Depression

Table 2 presents the bivariate analyses of the study variables by depression (yes/no). Married participants were at a lower risk of depression than divorced, single, or widowed participants (50.9% vs. 69.6%). Depression was far more prevalent among participants who were exposed to verbal bullying (65.5%) than non-exposed participants (48.6%), and among those exposed to physical bullying (81.8%) than those who were not exposed to physical bullying (54.3%). No differences in the rate of depression were found based on the exposure period to verbal bullying. Substantial differences in the prevalence of depression were found for all psychosocial indicators, as participants with low self-esteem were far more likely to suffer from depression than those with no indication of low self-esteem (66.4% vs. 20.8%). The same pattern emerged with regard to participants with social anxiety (89.5% compared to 42.3% among those not suffering from social anxiety) and those who reported social avoidance (71.8% compared to 45.5% among those not avoiding social situations). Finally, differences in the prevalence of depression were also observed by the presence of migraines (61.5% among those with migraines compared to 42.9% among those without migraines) and hypertension (70.0% among those with hypertension compared to 53.1% among those without hypertension).

### 3.3. Hierarchical Logistic Regression Models

Table 3 presents five hierarchical logistic models predicting the probability of depression. According to the first model, older age at time of claiming compensation is positively associated with depression (OR = 1.04, *p* < 0.05; 95% CI 1.00–1.08). Married participants (OR = 0.47, *p* < 0.01; 95% CI 0.28–0.80) and those who were irradiated at an older age (OR = 0.90, *p* < 0.05; 95% CI 0.83–0.98) were less likely to suffer from depression. As expected, the second model showed that being exposed to verbal and physical bullying increases the probability of developing depression (OR = 1.92, *p* < 0.05; 95% CI 1.19–3.10 and OR = 3.20, *p* < 0.01; 95% CI 1.01–10.12, respectively; model 2). Adding the psychosocial indicators in the third model revealed that low self-esteem and social anxiety greatly increase the probability of developing depression (OR = 4.36, *p* < 0.01; 95% CI 2.07–9.19 and OR = 8.03, *p* < 0.01; 95% CI 3.71–17.36, respectively). Social avoidance had no effect on the probability of developing depression (model 3). The fourth model indicated that migraines and hypertension are also positively associated with depression (OR = 2.48, *p* < 0.01; 95% CI 1.30–4.74 and OR = 2.79, *p* < 0.05; 95% CI 1.25–6.24, respectively; model 4). Finally, all other things being equal, the probability of developing depression was much lower for men than for women (OR = 0.38, *p* < 0.01; 95% CI 0.19–0.72; model 5). We found no evidence of the moderation effect (model not shown). That is, women are no more or less sensitive to any of the violence, psychosocial, and health measures considered compared to men.

## 4. Discussion

The aim of the current study was to examine gender differences in psychosocial and health outcomes of hair loss due to childhood irradiation for tinea capitis, and to identify the risk factors associated with depression in both men and women. We found significant gender differences in all four psychosocial indicators (i.e., low self-esteem, social anxiety, social avoidance, and depression), suggesting that women are at an increased risk of developing psychosocial symptoms. This may be due to the greater importance that women ascribe to their appearance than men [5,6]. The higher prevalence of psychosocial symptoms identified among the women reinforces previous findings on the effects of different types of alopecia, indicating lower psychological well-being [22] and QOL [23], as well as higher rates of depression [24] among women compared to men.

Interestingly, we did not find a significant gender difference in exposure to verbal and/or physical bullying because of hair loss. Both women and men were more likely to report having experienced verbal bullying than physical bullying. Both were also more likely to report having experienced verbal bullying during their childhood. This finding is in line with a previous study based on a community sample that showed a similar frequency of appearance-related teasing experiences in childhood and adolescence between men and women [44].

In regards to the physical health outcomes, a gender difference emerged only for hypertension, with a higher prevalence in women. Previous findings indicate that, in most countries, the prevalence of hypertension is higher in women than men after the age of 50 years [45], which was the age range of the participants included in our study at the time of filing the compensation claim. Therefore, we can assume that the higher prevalence of hypertension among the women in our study is not related to their hair loss.

Given the high prevalence of depression reported in our overall sample (56.2%) and the large gender difference that emerged for depression risk (i.e., women were nearly twice as likely as men to experience depression), we chose to focus our multivariate analysis on predicting the risk of depression and exploring the distinct contribution of gender to this outcome. This enabled us not only to identify the risk factors associated with depression among individuals with hair loss resulting from childhood irradiation for tinea capitis, but to determine whether the gender difference observed in depression risk is due to the higher prevalence of risk factors for depression among women or the effect of gender per se. The multivariate analysis revealed that, all other things being equal, women are much more likely than men to experience depression. These results clearly suggest that the gender difference in depression risk is not due to the higher prevalence of other risk factors among women, such as low self-esteem, social anxiety, and social avoidance, but rather due to the effect of gender per se. By shedding light on the role of gender in the development of depression among women with alopecia resulting from childhood irradiation for tinea capitis, our results add to earlier reports documenting depression among women with different types of alopecia [32,35,46,47,48] and higher rates of depression among women with androgenetic alopecia compared to men [24]. A plausible explanation for the important role that gender plays in the development of depression among women with alopecia is that hair symbolizes women’s fertility, femininity, sexuality, and attractiveness in society [7,9,10]. In this context, hair loss in women may result in an increased vulnerability to depression.

Furthermore, we found no evidence of a moderation effect; the associations between each of the risk factors included in our model and depression were similar among women and men. Eliminating the possibility that women are more sensitive than men to the risk factors considered here for depression further strengthens and confirms our conclusion that gender itself increases the risk of depression.

The results of our analysis reveal additional risk and protective factors for depression. With respect to the socio-demographic variables, being married and older at irradiation both decreased the risk of depression. The protective effect of marriage on mental health outcomes is consistent with previous findings in individuals with alopecia areata [49]. A possible explanation for the decreased risk for depression among participants who were irradiated at an older age is that a shorter exposure to appearance-related teasing may lessen its negative impact on mental health outcomes.

We found that experiences with verbal and, especially, physical bullying because of hair loss increased the risk of depression. In addition, controlling for the entire set of explanatory variables, low self-esteem and social anxiety had the strongest effect on depression, as indicated by their OR in the final model. The magnitude of their effect remained stable even after controlling for physical health measures, suggesting centrality of low self-esteem and social anxiety as major risk factors for depression. These results are consistent with prior results indicating that low self-esteem [50,51] and social anxiety [52] are significant risk factors for depression among additional populations.

With respect to physical health, we found that individuals with migraines, hypertension, and cancer were more likely than their counterparts to suffer from depression, with somewhat larger effects for migraines and hypertension. These results support previous findings linking migraines [53,54,55] and hypertension [56,57,58] with an increased risk of developing depression.

This study has two major limitations. Firstly, the disadvantages inherent to a retrospective review of medical records include missing data, difficulty interpreting the documented information, and variability in the quality of the documented information [59]. The study also has the limitation of extracting self-reported data from the medical files that were used to apply for medical compensation, possibly allowing exaggeration to have occurred. The use of self-reported data, however, also reflects a major strength of this study, because this information provides insight into the subjective experiences of women and men living with persistent alopecia from childhood.

## 5. Conclusions

The results of the present study indicate that the gender difference we identified in depression risk is not due to the higher prevalence of other risk factors among women, such as low self-esteem, social anxiety, and social avoidance, but rather due to the effect of gender per se. These results suggest that healthcare professionals supporting individuals with a history of tinea capitis should be especially alerted to women with significant hair loss resulting from the irradiation treatment they underwent in childhood given the long-term risk for the development of depression. Our results also highlight the need for healthcare professionals to take into account these women’s increased risk of developing low self-esteem, social anxiety, and social avoidance.

The contribution of this study is two-fold. First, it adds to the limited empirical evidence on gender differences in hair loss-related depression. Second, its contribution goes beyond hair loss resulting from childhood irradiation for tinea capitis, as it provides insight into the differential effects of persistent hair loss on a range of psychosocial symptoms in women and men. Our results indicate that hair loss resulting from childhood irradiation is a major and long-term risk factor for depression, and not just an aesthetic issue, particularly in women. These results suggest that women are more vulnerable than men to the psychosocial effects of irreversible aesthetic damages resulting from medical treatments.

## Figures and Tables

**Table 1:** Bivariate Analyses of the Study Variables by Gender (N = 322).

	N	Overall (*n* = 322)	Female (*n* = 217)	Male (*n* = 105)	
		Mean (SD)/%	Mean (SD)/%	Mean (SD)/%	t/Chi-Square
**Background Variables**					
**Age at filing a compensation claim**		57.99 (7.02)	57.78 (7.19)	58.43 (6.68)	−0.77
43–52	78	24.2	25.8	21.0	3.28
53–61	159	49.4	48.4	51.4	
62–70	65	20.2	18.4	23.8	
71–80	20	6.2	7.4	3.8	
**Marital status**					11.70 **
Married	230	71.4	65.4	83.8	
Other (divorced single widow)	92	28.6	34.6	16.2	
**Years of education**		7.95 (4.07)	7.57 (4.17)	8.69 (3.80)	−2.03 *
0–8	229	71.1	73.3	66.7	1.67
9–11	37	11.5	11.1	12.4	
12+	56	17.4	15.7	21.0	
**Employment at the time of the claim**					15.59 **
Yes	241	74.8	68.2	88.6	
No	81	25.2	31.8	11.4	
**SES factor score**		0.00 (1.00)	−0.12 (1.03)	0.26 (.86)	−3.37 **
**Age at radiation**		8.38 (3.39)	8.00 (3.37)	9.16 (3.30)	−2.93 **
3–5	79	24.5	28.1	17.1	9.46 **
6–10	153	47.5	48.8	44.8	
11+	90	28.0	23.0	38.1	
**Place of radiation**					
Israel	168	55.1	52.6	60.4	7.60 *
North Africa	111	36.4	35.9	37.5	
Europe and other	26	8.5	11.5	2.1	
**Exposure to verbal and physical bullying**					
**Verbal bullying**					2.25
Yes	145	45.0	47.9	39.0	
No	117	55.0	52.1	61.0	
**Physical bullying**					0.31
Yes	22	6.8	7.4	5.7	
No	300	93.2	92.6	94.3	
**Period of exposure to verbal bullying**					1.39
Childhood	88	60.7	57.7	68.3	
Adulthood	27	18.6	20.2	14.6	
Childhood and adulthood	30	20.7	22.1	17.1	
**Psychosocial indicators**					
**Low self-esteem**					9.01 **
Yes	250	77.6	82.5	67.6	
No	72	22.4	17.5	32.4	
**Social anxiety**					19.59 **
Yes	95	29.5	37.3	13.3	
No	227	70.5	62.7	86.7	
**Social avoidance**					8.04 **
Yes	131	40.7	46.1	29.5	
No	191	59.3	53.9	70.5	
**Depression**					
Yes	181	56.2	66.8	34.3	30.43 **
No	141	43.8	33.2	65.7	
**Physical health indicators**					
**Migraine**					0.12
Yes	231	71.7	72.4	70.5	
No	91	28.3	27.6	29.5	
**Hypertension**					6.84 **
Yes	60	18.6	22.6	10.5	
No	262	81.4	77.4	89.5	
**Diabetes**					0.58
Yes	63	19.6	20.7	17.1	
No	259	80.4	79.3	82.9	
**Tumors**					1.13
Yes	130	40.4	42.4	36.2	
No	192	59.6	57.6	63.8	

Note. * *p* < 0.05; ** *p* < 0.01.

**Table 2:** Bivariate analyses of the study variable by depression (N = 322).

Depression			
	Yes 56.2% (*n* = 181)	No 43.8% (*n* = 141)	T/Chi-Square
	%/Mean (SD)	%/Mean (SD)	
**Background Variables**			
**Age at filing a compensation claim**	58.35 (7.05)	57.53 (6.97)	−1.04
**Marital status**			9.33 **
Married	50.9	49.1	
Other (divorced single widow)	69.6	30.4	
**Years of education**			1.45
0–8	54.1	45.9	
9–11	59.5	40.5	
12+	63.5	37.5	
**Employment at the time of the claim**			0.01
Yes	56.4	43.6	
No	55.6	44.4	
**SES factor score**	0.04 (1.01)	−0.05 (0.97)	−0.84
**Age at radiation**	8.09 (3.21)	8.74 (3.53)	1.70
**Exposure to verbal and physical bullying**			
**Verbal bullying**			9.28 **
Yes	65.5	34.5	
No	48.6	51.4	
**Physical bullying**			6.29 *
Yes	81.8	18.2	
No	54.3	45.7	
**Verbal bullying period**			0.66
Only In childhood	63.6	36.4	
Only In adulthood	70.4	29.6	
In childhood and in adulthood	70.0	30.0	
**Psychosocial indicators**			
**Low self-esteem**			47.15 **
Reported	66.4	33.6	
Not reported	20.8	79.2	
**Social anxiety**			60.57 **
Yes	89.5	10.5	
No	42.3	57.7	
**Social avoidance**			21.68 **
Yes	71.8	28.2	
No	45.5	54.5	
**Physical health indicators**			
**Migraine**			9.19 **
Yes	61.5	38.5	
No	42.9	57.1	
**Hypertension**			5.69 *
Yes	70.0	30.0	
No	53.1	46.9	
**Diabetes**			0.20
Yes	58.7	41.3	
No	55.6	44.4	
**Tumors**			1.27
Yes	60.0	40.0	
No	53.6	46.4	

Note. * *p* < 0.05; ** *p* < 0.01.

**Table 3:** Hierarchical logistic regression models: Prediction of depression (N = 322).

Variables	Model 1		Model 2		Model 3		Model 4		Model 5	
	B (SE)	OR	B (SE)	OR	B (SE)	OR	B (SE)	OR	B (SE)	OR
Age at Filing a Compensation Claim	0.04 * (0.02)	1.04	0.04 * (0.02)	1.04	0.06 * (0.02)	1.06	0.06 * (0.02)	1.06	0.06 * (0.02)	1.07
Marital Status										
Other (Divorced Single Widow) (ref)										
Married	−0.74 ** (0.26)	0.47	−0.74 ** (0.27)	0.47	−0.62 * (0.30)	0.53	−0.56 (0.32)	0.56	−0.41 (0.33)	0.66
SES Factor Score	0.11 (0.11)	1.12	0.12 (0.11)	1.13	0.04 (0.13)	1.04	0.02 (0.14)	1.02	0.14 (0.14)	1.15
Age at Radiation	−0.10 * (0.04)	0.90	−0.11 ** (0.04)	0.89	−0.10 * (0.04)	0.90	−0.09 0.05	0.90	−0.07 (0.05)	0.92
Verbal Bullying (yes = 1)			0.65 ** (0.24)	1.92	−0.09 (0.30)	0.91	−0.05 (0.31)	0.95	−0.07 (0.32)	0.93
Physical Bullying (yes = 1)			1.16 * (0.58)	3.20	1.02 (0.62)	2.77	1.04 (0.65)	2.82	1.23 (0.69)	0.88
Low Self-Esteem (yes = 1)					1.47 ** (0.38)	4.36	1.63 ** (0.40)	5.12	1.59 ** (0.41)	4.92
Social Anxiety (yes = 1)					2.08 ** (0.39)	8.03	2.24 ** (0.41)	9.39	2.12 ** (0.41)	8.35
Social Avoidance (yes = 1)					0.09 (0.30)	1.10	0.17 (0.32)	1.19	0.14 (0.33)	1.15
Migraine (yes = 1)							0.91 ** (0.33)	2.48	0.96 ** (0.33)	2.62
Hypertension (yes = 1)							1.02 * (0.41)	2.79	0.85 * (0.41)	2.36
Diabetes (yes = 1)							−0.18 (0.38)	0.83	−0.15 (0.38)	0.85
Tumors (yes = 1)							0.58 * (0.30)	1.79	0.56 (0.31)	1.76
Gender (man = 1)									−0.96 ** (0.32)	0.38
Intercept	−0.77 (1.09)	0.46	−1.46 (1.13)	0.23	−3.65 ** (1.32)	0.02	−5.29 ** (1.49)	0.00	−5.33 ** (1.52)	0.00
Chi Square	17.72 **		32.74 **		107.85 **		130.13 **		139.19 **	
Pseudo R^2^	0.072		0.130		0.381		0.446		0.470	

Note. * *p* < 0.05; ** *p* < 0.01.

## Data Availability

The data presented in this study are available on request from the corresponding author. The data are not publicly available due to ethical restrictions.
